# Cutaneous Leishmaniasis Emergence in Southeastern Mexico: The Case of the State of Yucatan

**DOI:** 10.3390/tropicalmed7120444

**Published:** 2022-12-17

**Authors:** Elsy B. Canché-Pool, Jesús A. Panti-May, Hugo A. Ruiz-Piña, Marco Torres-Castro, Francisco J. Escobedo-Ortegón, Paulino Tamay-Segovia, Selene Blum-Domínguez, Jimmy R. Torres-Castro, Enrique Reyes-Novelo

**Affiliations:** 1 Centro de Investigaciones Regionales “Dr. Hideyo Noguchi”, Universidad Autónoma de Yucatán, Av. Itzaes No. 490 x 59, Col. Centro, Mérida 97200, Mexico; 2 Centro de Investigaciones Biomédicas, Universidad Autónoma de Campeche, Av. Agustín Melgar s/n x 20 y Juan de la Barrera, Col. Buenavista, Campeche 24039, Mexico; 3 Servicios de Salud de Yucatán, Dirección de Prevención y Protección de la Salud, Calle 72 No. 463 x 53 y 55 Col. Centro, Mérida 97000, Mexico

**Keywords:** *Leishmania*, Phlebotominae, mammals, reservoir, sand flies

## Abstract

Environmental changes triggered by deforestation, urban expansion and climate change are present-day drivers of the emergence and reemergence of leishmaniasis. This review describes the current epidemiological scenario and the feasible influence of environmental changes on disease occurrence in the state of Yucatan, Mexico. Relevant literature was accessed through different databases, including PubMed, Scopus, Google, and Mexican official morbidity databases. Recent LCL autochthonous cases, potential vector sandflies and mammal hosts/reservoirs also have been reported in several localities of Yucatan without previous historical records of the disease. The impact of deforestation, urban expansion and projections on climate change have been documented. The current evidence of the relationships between the components of the transmission cycle, the disease occurrence, and the environmental changes on the leishmaniasis emergence in the state shows the need for strength and an update to the intervention and control strategies through a One Health perspective.

## 1. Introduction

Leishmaniasis is a vector-borne zoonotic disease caused by several species of *Leishmania* parasites. Its transmission cycle involves several vertebrate mammals (primary reservoirs and secondary or accidental hosts) and vector sandfly species (Psychodidae: Phlebotominae) [1]. It is a significant health problem in many world areas and continues to spread to new areas in endemic and non-endemic countries. In 2020, 98 countries were considered endemic to leishmaniasis [2]. Its clinical expressions vary, but the most frequent are cutaneous leishmaniasis (CL), mucocutaneous leishmaniasis (ML) and visceral leishmaniasis (VL) [3].

All clinical forms occur in Mexico, but localized cutaneous leishmaniasis (LCL) is the most frequently occurring since 99% of new annually reported cases belong to this clinical form [4]. The main causal agent of LCL in the country is *Leishmania (Leishmania) mexicana*. The recognized primary vector for this parasite species is the sandfly *Bichromomyia olmeca olmeca*. The rodents of the genera *Heteromys*, *Nyctomys*, *Ototylomys*, *Sigmodon* and *Peromyscus* are the reservoir hosts of *Leishmania* parasites in several active transmission areas [1,5,6].

In 1912, Seidelin published the first description of the disease in latex (the raw material for chewing gum) collecting workers from the Yucatan Peninsula (comprised of the states of Quintana Roo, Campeche, and Yucatan). Ulcerative lesions developed predominantly in their ears and became known as “Chiclero’s ulcers” [7]. Latex harvest was one of the main activities in forested areas, covering 90% of Quintana Roo lands, two-thirds of Campeche’s and a small strip in the south of Yucatan state. Therefore, the disease prevailed in Campeche and Quintana Roo, and infection was related to sylvatic environments [7,8].

Until today, the sylvatic regions of Campeche, Quintana Roo, Tabasco and Chiapas, all in south-southeast Mexico, continue to be the main endemic areas of transmission, where *Leishmania* parasites, vector sandflies and mammal reservoirs have been extensively studied [1,4,9,10,11].

Historically, the northern area of the peninsula occupied by the state of Yucatan is not endemic for leishmaniasis although the state is classified as a vulnerable and receptive area for *Leishmania* transmission [1]. Therefore, recent autochthonous cases suggest new local transmission demanding an updated analysis of the epidemiological situation of the disease, including human cases, sandfly vectors, hosts/reservoirs and environmental factors that may favor the emergence of new transmission foci. In this review we analyze and describe relevant literature about human cases of cutaneous leishmaniasis, *Leishmania* parasite species, vector sandfly species, vertebrate hosts and environmental disturbances in Yucatan. Information was accessed through different databases, including PubMed, Scopus, Google and morbidity official databases from Yucatan and Mexico from the Mexican Health Ministry.

## 2. Human LCL in Yucatan State and Mexico

### 2.1. Historical Records

Although leishmaniasis was first described in Mexico in 1912, mandatory epidemiological records by the Mexican Ministry of Health began only at the end of 1985 [9]. From this year until 2014 (29 years), less than 0.5% of annual cases (26 cases) were registered in Yucatan, while the highest numbers for the country were recorded in Campeche (2414) and Quintana Roo (5525) in the same period [9]. Besides, immunosurveys conducted in a municipality located in the south region of Yucatan showed 17% of people leishmanin skin test positive, probably due to subclinical infection [12]. The leishmanin skin test (LST; formerly known as the Montenegro skin test) is performed via intradermal injection of *Leishmania* antigens to induce and visualize the adaptive immune response in individuals who have been previously infected with *Leishmania*. The test had been a useful tool in epidemiological studies to monitor exposure and immunity to *Leishmania* as well as in vaccine studies as a surrogate marker of immunity [13]. Therefore, the study results indicate that a significant percentage of people who lived in the south region of Yucatan, close to highly endemic areas, developed an immune response against *Leishmania* parasites. From 1985 to 2014, no evidence of local leishmaniasis was found in the Yucatan state.

### 2.2. Current Situation

**Figure 1:**
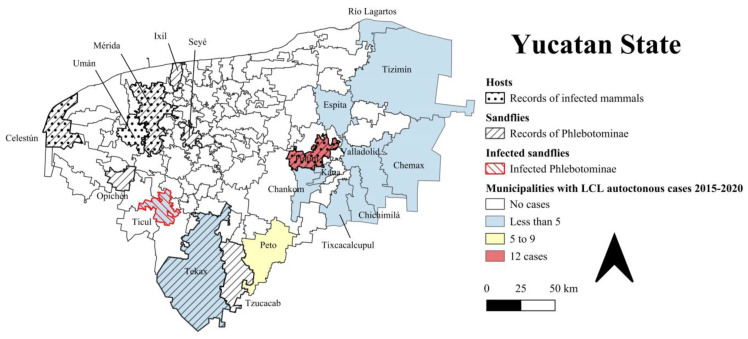
Geographical distribution of mammal hosts infected by *Leishmania*, vector sandfly species, and autochthonous LCL human cases in Yucatan state. Dotted boxes: municipalities with record of *Leishmania* infected mammals [18,19,20,21]. Boxes with lines: municipalities with vector sandfly species reports [22,23,24,25]. Color boxes: municipalities with diagnosed autochthonous cases of LCL from 2015 to 2020.

From 2015 to 2020 in Yucatan, the number of LCL cases registered per year increased up to 2.9% (36 cases in the period) [9]. In this time lapse, LCL cases were diagnosed in areas from 13 municipalities with no previous records of any outbreak and in patients with no travel history to endemic regions (accordingly with operational definitions of Mexican Health Services) [9,14,15,16]. Only three municipalities are in the southern region, namely, Tekax, Peto and Ticul, while Tinum, Kaua, Chankom, Valladolid, Chemax, Tekom and Chichimila are in the eastern region; Espita and Río Lagartos are in the northeastern region; and Kinchil is in the western region of the state (Figure 1).

In a recent study, *L. mexicana* was identified with polymerase-chain reaction test as the causal agent of the disease in patients from Peto, Tekom, Espita, Tinum, Chichimila, Río Lagartos, and Ticul [16]. According to revised reports, leishmaniasis cases were diagnosed through parasitological testing with Giemsa-stained smears and treated with meglumine antimoniate through the programs promoted by the State and Mexican Ministry of Health [14,15,16]. Cases were predominantly males ranging from 14 to 77 years who developed lesions on the ear, face, upper limbs, and chest. The majority of human LCL cases are associated with activities in field crops and forests, suggesting a sylvatic transmission cycle as in highly endemic regions of Campeche and Quintana Roo [16,17]. Also, subclinical infections were reported in people from municipalities with recent outbreaks such as Tinum, where 13% of 47 individuals without typical lesion or scar were positive to the leishmanin skin test [17]. This suggests that (1) biological and ecological factors may allow the transmission and spread of the disease in new areas of the state and (2) the disease may be underestimated in Yucatan.

## 3. Vector Sandfly Species

The vectors of *Leishmania* parasites are hematophagous dipterans of the family Psychodidae, subfamily Phlebotominae. In the Americas, several genera concentrate species exclusively distributed on the continent. However, the species of public health relevance are found in the genera *Lutzomyia*, *Bichromomyia* and *Psychodopygus* due to their demonstrated capacity as vectors involved in the transmission of the different *Leishmania* species [26].

In Mexico, *Bi. olmeca olmeca* is recognized as the primary vector responsible for the transmission of *Leishmania* to humans. However, *Lu. cruciata* has also been incriminated as a potential vector, mainly due to its anthropophilic nature and abundance in endemic areas. Furthermore, other species, such as *Psathyromyia (Psathyromyia*) *shannoni*, *Psychodopygus panamensis* and *Nyssomyia ylephiletor*, have been recently found involving *L. mexicana* in the state of Campeche [1,6].

In Yucatán, the presence of 15 Phlebotominae species has been documented: *Brumptomyia hamata*, *Br. mesai*, *Dampfomyia* (*Coromyia*) *beltrani*, *Da.* (*Cor*.) *deleoni*, *Lutzomyia* (*Lutzomyia*) *longipalpis*, *Lu.* (*Tricholateralis*) *cruciata*, *Bi*. *olmeca olmeca*, *Pintomyia* (*Pifanomyia*) *serrana*, *Psathyromyia* (*Forattiniella*) *carpenteri*, *Pa* (*Psa*.) *cratifer*, *Pa*. (*Psa*.) *shannoni*, *Pa*. (*Psa*.) *undulata*, *Micropygomyia* (*Coquillettimyia*) *chiapanensis*, *Mi*. (*Micropygomia*) *cayennensis maciasi* and *Mi*. (*Sauromyia*) *trinidadensis* [22,23,24].

Current geographic records of Phlebotomines show a wide distribution into the Yucatan territory, where Phlebotominae vectors would not be expected to occur in northern municipalities with high population densities in urban settings like Mérida, Umán or high population affluence like Celestún (Figure 1); probably because the study of the Phlebotominae fauna in Yucatan has been historically sporadic due to the absence of leishmaniasis cases. To our knowledge, only the study of Pérez-Blas et al. has recorded molecular evidence of *Lu. cruciata* and *Pa. shannoni* carrying *L. mexicana* in a locality from Ticul municipality [25]. Formal studies on the abundance and distribution of these insects have been limited in the last 15 years, where a couple of studies focused on specific localities, which shows a noticeable need to study in greater detail the populations of species of medical importance and the changes in their distribution and abundance derived from anthropogenic transformation and environmental variation since these are factors that directly affect the establishment and maintenance of their populations [22,24,27].

## 4. Vertebrate Hosts

In the Americas, more than 80 species of several mammal orders have been reported infected with *Leishmania* parasites, such as opossums (Didelphimorphia), anteaters, sloths (Pilosa), armadillos (Cingulata), mice, squirrels (Rodentia), dogs, jaguars (Carnivora), bats (Quiroptera), horses (Perissodactyla), monkeys and humans (Primates) [28,29]. However, not all these hosts play the same role in the transmission of *Leishmania*. Most mammals may act as “dead-end hosts”, which means that they develop low levels of parasitaemia and cannot pass the parasites on to susceptible sand flies. In contrast, few mammalian species can act as reservoir hosts given that they can maintain sufficient values of parasitaemia and for longer periods to efficiently infect vectors [28].

In Yucatan, the infection with *Leishmania* has been investigated in domestic dogs and cats. In Celestún, Longoni et al. found that the sera of 29 (52.7%) dogs were positive for *Leishmania* spp. [18]. Arjona-Jiménez et al. also found a similar frequency (50.5%, 110/218) of *Leishmania*-positive dogs in the city of Mérida [19]. Longoni et al. identified specific antibodies against *Leishmania* spp. in 6 (54.5%) and 18 (25%) cats from the cities of Umán and Mérida, respectively [20]. This serological evidence indicates an exposure of these animals to the parasites and the likelihood of vectorial transmission in these localities close to Kinchil (ca. 20–44 km), where a case of human leishmaniasis was reported. (Figure 1).

There is scientific evidence in Campeche and other regions of Mexico and Latin America that highlight the role of rodents, bats, and opossums as reservoir hosts of *Leishmania* [29,30,31], suggesting potential participation in leishmaniasis foci on Yucatan. In Campeche, studies on the sylvatic cycle of *Leishmania* have reported six wild rodents (*Heteromys gaumeri*, *Peromyscus yucatanicus*, *Oryzomys melanotis*, *Ototylomys phyllotis*, *Reithrodontomys gracilis* and *Sigmodon toltecus*) infected with the *Leishmania* parasites [30,31]. Data on seasonality of infection suggest that *H. gaumeri*, *P. yucatanicus* and *O. phyllotis* may be the primary reservoir hosts of *Leishmania* in Campeche [32]. These rodent species are widely distributed in Yucatan. Occurring in tropical forests, grassland, and cornfields, however, their abundances vary in response to rain, food availability, conservation status of tropical forests, topography, and other factors [33]. *Heteromys gaumeri* is abundant in southern Yucatan, whereas *P*. *yucatanicus* and *O*. *phyllotis* predominate in north-central areas of the state [34,35,36]. Moreover, *P. yucatanicus* and *S. toltecus* may visit backyards of rural households associated with secondary forests [37,38]. The presence of *Leishmania* in wild rodents has only been investigated in Tinum, Yucatan where *H. gaumeri* and *O. phyllotis* were found infected by the parasites [21].

Berzunza-Cruz et al. provided the first evidence of *Leishmania* infection in 13 bat species from the Chiapas–Tabasco ecoregion and Jalisco, Mexico [39]. Chiroptera is the most diverse order of mammals in Yucatan with an estimated 43 species belonging to seven families [40]. Eight species of bats (*Pteronotus psilotis*, *Artibeus jamaicensis*, *A. lituratus*, *Dermanura phaeotis*, *Carollia sowelli*, *Desmodus rotundus*, *Glossophaga mutica* and *Sturnira parvidens*) that were found naturally infected by *Leishmania* also exist in Yucatan [39]. Most of them belong to the family Phyllostomidae, except for *P. psilotis*, which belongs to the family Mormoopidae. Phyllostomidae is the most diverse family of bats in Yucatan [40]. Members of this family are frugivorous, insectivorous, nectarivorous, carnivorous and hematophagous (although some species have mixed diets) and inhabit a variety of natural sites, such as caves, rock crevices, foliage and hollow trees. They commonly occupy artificial sites like buildings and householding surroundings [41]. The evidence points out a noticeable research gap on *Leishmania* bat infection in the region.

*Leishmania* infection in opossums of the genus *Didelphis* has been reported in several countries of Latin America in both sylvatic and domestic habitats [42,43,44,45]. In Yucatan, this genus is represented by two species, *Didelphis virginiana* and *D. marsupialis*. The first species abound in the northern area of the state, whereas the later species predominates in central-south areas of the state [46]. Opossums of the genus *Didelphis* can adapt to human-modified landscapes due to their ability to exploit a wide range of resources and environments [47]. Particularly, *D. virginiana* is a species that has been extensively studied in Yucatan and reported as a common visitor of the peridomicile of urban and rural areas where they find food (e.g., fruits, chicks, organic waste) and shelters (e.g., roofs of houses, hollow trees, and piles of rocks) [46], but surprisingly, an opossum infection with *Leishmania* has not been reported in Mexico.

## 5. Impact of Environmental Factors on the Emergence of Leishmaniasis

The ecology and epidemiology of vector-borne diseases, such as leishmaniasis, are affected by the interrelations between the pathogen, vectors, hosts (human and non-human animals) and their associations with the environment. Therefore, physical, biological, and socioeconomic events that affect this interaction can influence its transmission and spread [48]. Environmental changes, population mobility, socioeconomic conditions and malnutrition are major risk factors for leishmaniasis [3]. We highlight the role of environmental changes caused by deforestation, urbanization, and climate change due to their direct impact on the ecosystems and their relationship with the transmission and spread of leishmaniasis. These factors also have been related to the disturbance of ecosystems in Yucatan and may influence the transmission of the disease; however, we do not rule out that other factors, such as socioeconomic policies and malnutrition, may also affect its spread or emergence in previously un-recorded areas.

### 5.1. Deforestation and Urbanization

The human incursion into forested endemic areas and changes in urbanization influences the incidence of leishmaniasis [3]. Habitat change by deforestation, extensive land use, agricultural development and alterations in water storage and irrigation habits provide new niches for arthropod vectors [49,50]. Deforestation and transformation of forests to grazing lands, agricultural areas, human settlements, or open areas result in significant alterations in the environment and changes in the composition of vector communities and, therefore, the introduction of new pathogens [48]. Forest-related activities, such as mining and logging, have been associated with increased exposure to the vectors of yellow fever, malaria and leishmaniasis [51]. The human population growth and massive urbanization are associated with displacement of people from rural areas to urban centers, creating new contacts and interactions in the new densely populated environment [48,50]. For instance, visceral leishmaniasis in Brazil was predominantly a rural disease; however, recent outbreaks have occurred in several Brazilian cities due to massive population movement from rural areas to cities [51]. Also, some forms of cutaneous leishmaniasis typically occur as epidemics in densely populated cities in central and western Asia [51].

In Mexico, ecosystems’ degradation by human activities has been one of the great environmental issues. Although this problem has been present throughout the country, it has been more intense in the south and southeast due to its greater biodiversity and conserved habitats [52].

In Yucatán, deforestation and land use for agricultural activities have had the greatest impact on ecosystems. From 1976 to 2000, crop areas increased, and the forest cover was lost due to its use for livestock and agriculture [53]. The most affected regions are located around Mérida and the east of the state, where the municipalities of Río Lagartos, Tizimín, Calotmul, Temozón, Valladolid and Chemax are located [52,53]. The construction of harbor infrastructure (breakwaters, dams, ports) and roads has fragmented the wetland areas (mangroves, swamps), beaches and dunes, which can affect the habitats and ecological interactions in these sites. The most environmentally affected areas are those adjacent to the access roads of main ports, namely Celestún, Sisal, Chuburná, Chelém, Yucalpetén, Progreso, Telchac, Dzilam Bravo and Río Lagartos [53].

Durán-García et al. [54] summarize that population growth and urbanization have also contributed to the modification of natural systems in the Yucatan. In 2005, there were 1,818,948 inhabitants in the state; more than one million compared to 1970. In addition to this, in recent decades, the population has been concentrated in the metropolitan area including the city of Merida (the capital of the state) and the outlying municipalities, especially Umán, Progreso and Kanasín. This area brings together almost 1 million inhabitants, while another 800,000 are dispersed throughout the rest of the state territory. This scenario has been increasing and, according to the recent population census (2020), Yucatán was inhabited by 2,320,898 people [55].

The most recent evidence of the potential effect that deforestation and urbanization might have on leishmaniasis transmission in Yucatan was the finding of a potential emergent focus in Tinum municipality. Human cases, two rodent species with *Leishmania* infection evidence [17,21] and sandflies were recorded in sites under different land use of fragmented tropical deciduous forests mixed with crops and urban expansion [56]. The studies document that people use areas where infected animals and sandflies converge and, even when sandflies’ abundances were greater in forested areas, generalist, and colonialist *Lu. cruciata* might pose a potential risk for leishmaniasis transmission expansion to non-forested areas. The occurrence in human setting surroundings is crucial for future understanding of the ecology of this sandfly and its interaction with *Leishmania* [56].

### 5.2. Climate Change

Temperature and rainfall are environmental factors that influence the transmission dynamics and geographic spread of vector-borne diseases. Temperature affects vector reproduction, metabolism and survival, pathogen replication and vector and host distribution, while rainfall can determine the suitability of habitats for vectors and hosts through increased breeding sites [57]. Meteorological events, such as flooding and heat waves, can result in the destruction of suitable habitats for vectors and hosts of *Leishmania* [57]. A shift in sandflies vector abundance and distribution has been reported from south to north Europe which has been attributed to changes in the climatic conditions as modeled through ecological niche [58]. Sand flies vector species competent for transmitting *Leishmania* parasites were recently found for the first time in Belgium and Germany, creating new transmission risk scenarios in countries that are currently free of the disease. The same displacement of competent sand flies was also reported in the southern hemisphere, from north to south Argentina due to an increase of temperatures in more temperate regions of this country [59].

Mexico, specifically the Yucatan Peninsula, and Central America have been identified as areas particularly vulnerable to adverse climate change consequences. From 1951 to 2017 the temperature in Mexico has increased by 0.7 °C without variation in precipitation regimes. However, the wet seasons (June–November) have become wetter and the dry seasons (December–May) drier [60]. Projections for Mexico suggest that precipitation will decrease, with the largest decreases in summer rainfall (~13%) in southern Mexico. Conversely, temperatures will increase between 2 and 4 °C by the end of the century [59].

Projections on the climate variation in Yucatan suggest that temperature will increase between 0.5 and 0.8 °C for the 2010–2030 period, between 0.5 °C and 1.8 °C for 2040–2060 and between 0.6 and 2.8 for the 2070–2090 period [61]. The increase may be more evident in the northwest and west of the state. Maximum temperatures between 33 and 37.4 °C would be reached in 80% of the state surface with the east, northeast and southeast of the state being the most affected areas, particularly the municipalities of Tizimín, Chemax, Temozon, Valladolid, Chichimila, Calotmul, Espita, Uayma, Tixcacalcupul, Chikindzonot, Peto, Kahua, Tekom, Cuncunul, Río Lagartos, San Felipe, Panaba y Sucilá. Besides, precipitation may decrease between 1 and 15.3% at the end of the 21st century and affect the north more than the south of the state [61]. Climate change effects and the increase in extreme weather events, combined with a growing population are likely to have adverse effects on society and ecosystems.

The future climatic changes are expected to have a significant impact on the biodiversity and the distributions of plants and animals. In the Yucatan Peninsula, a recent study estimated the potential effects of climatic change on the distribution of three small rodents (*P. yucatanicus*, *H. gaumeri* and *Otonyctomys hatti*) based on ecological niche modeling [33]. Based on this model, in the future scenarios a slight increase of the suitable areas for the three rodent species is predicted. These probable scenarios also indicate that potential reservoir hosts such as *P. yucatanicus* and *H. gaumeri* may increase their distribution and the transmission risk of zoonoses, among them, leishmaniasis.

## 6. Conclusions

Leishmaniasis is a neglected public health problem, mainly for tropical and subtropical areas of the world, despite current control initiatives. Environmental changes caused by anthropogenic activities that disturb natural ecosystems will continue influencing the emergence and reemergence of the disease, particularly in tropical areas. The evidence reviewed here highlights the occurrence of human leishmaniasis cases in several municipalities of Yucatan, where local transmission of the disease had not previously occurred.

Very likely, we are witnessing the spread of leishmaniasis from hyperendemic areas to vulnerable and receptive areas of the peninsula and the emergence of new transmission foci in the Yucatan state. Global environmental changes are occurring in Yucatan. Deforestation and urban expansion will reach areas projected to have medium to severe environmental changes in the following decades, driving vector and reservoirs ecology changes, raising the risk of disease emergence in unexpected localities.

Further research must look for the forecasting and evaluation of scenarios of environmental changes to be able to predict the effects on the emergence of leishmaniasis in the state. Given that control strategies are limited, prevention and action through the One Health approach could lead to sustainable acts against disease emergence and increasing risk.

The One Health approach proposes to address health problems from the different aspects involved in their occurrence. From this perspective, the emergence of leishmaniasis foci in Yucatan shows the need to strengthen a multisectoral approach, consisting primarily in the joint work of different sectors to address the health problem [62]. The information described in this review shows how the government sector, through the Mexican health authorities and the academic sector, has begun the task of jointly addressing the identification, documentation, and act on the emergence of new outbreaks, each from their areas of responsibility and capacities for action. There are new windows of opportunity for the integration of the environmental and food production sectors, seeking to expand the prevention and response capacity in a coordinated manner since the scenario favors the appearance of new outbreaks and the potential establishment of new transmission foci.

## Data Availability

Not applicable.
